# *Bacillus coagulans* MZY531 alleviates intestinal mucosal injury in immunosuppressive mice via modulating intestinal barrier, inflammatory response, and gut microbiota

**DOI:** 10.1038/s41598-023-38379-0

**Published:** 2023-07-10

**Authors:** Zhongwei Zhao, Manqing Sun, Xinmu Cui, Jiaxin Chen, Chunhong Liu, Xuewu Zhang

**Affiliations:** 1 Medical College, Yanbian University, Yanji, 133002 Jilin People’s Republic of China; 2 College of Special Education, Changchun University, Changchun, 130022 People’s Republic of China

**Keywords:** Microbiology, Zoology, Gastroenterology

## Abstract

*Bacillus coagulans* has a potential role in improving intestinal injury. However, the specific mechanism is still unclear. In this study, the protective effect of *B. coagulans* MZY531 on intestinal mucosa injury in cyclophosphamide (CYP)-induced immunosuppressed mice were investigated. The results indicated that the immune organ (thymus and spleen) indices of *B. coagulans* MZY531 treatment groups were significantly increased compared to the CYP group. *B. coagulans* MZY531 administration promotes the expression of immune proteins (IgA, IgE, IgG, and IgM). *B. coagulans* MZY531 could upregulate the ileum levels of IFN-γ, IL-2, IL-4, and IL-10 in immunosuppressed mice. Moreover, *B. coagulans* MZY531 restores the villus height and crypt depth of the jejunum and alleviates injury of intestinal endothelial cells caused by CYP. Furthermore, the western blotting results showed that *B. coagulans* MZY531 ameliorated CYP-induced intestinal mucosal injury and inflammatory via up-regulates the ZO-1 pathway and down-regulates the expression of the TLR4/MyD88/NF-κB pathway. After treatment with *B. coagulans* MZY531, the relative abundance of *Firmicutes phylum* was dramatically increased, as well as the genera of *Prevotella* and *Bifidobacterium*, and reducing harmful bacteria. These findings suggested that *B. coagulans* MZY531 has a potential immunomodulatory activity on chemotherapy-induced immunosuppression.

## Introduction

Normal gastrointestinal function is essential for the absorption of nutrients, while gastrointestinal changes can lead to serious defects in the intestinal barrier and gastrointestinal diseases^1,2^. Tight junction proteins, including ZO-1, occludin, and claudin-1, constitute intestinal epithelial cells as a physical barrier, and their complex interactions maintain the integrity of the intestinal barrier and reduce intestinal permeability^3^. Intestinal leakage can activate immune cells to secrete inflammatory cytokines, which in turn increases intestinal permeability and causes systemic inflammation^4^. In addition, microorganisms in the gastrointestinal tract can also damage the intestinal barrier function by promoting mucosal reaction, reducing the expression of tight junction proteins. After reaching the blood, bacteria can trigger inflammation by binding TLR4 receptors expressed on the liver through the portal vein^5,6^.

Cyclophosphamide (CYP) was an alkylated anticancer agent which was widely used in the treatment of various cancers. However, long-term treatment of CYP may could result various side effects such as acute cytotoxicity, immunosuppression, and gastrointestinal mucosal barrier damage. To improve this situation, some protective agents are increasingly being used to alleviate adverse side effects in chemotherapy patients. Certain anti-parasitics have also been reported to have an immunomodulatory activity such as levamisole. Levamisole is a drug widely used to enhance the immunity of various human diseases, including leprosy, rheumatoid arthritis, and in adjuvanted therapy of colorectal cancer. In recent years, more and more attention has been paid to alleviating CYP induced immunosuppression based on gut microbiota regulation. In 2013, Viaud et al*.* demonstrated that CYP alters microbiota composition in the small intestine and induces the translocation of selected species of Gram-positive bacteria into secondary lymphoid organs^7^. A few years later, Xie et al*.* discovered that *Lactobacillus plantarum* intervention could protect the intestinal mucosal injury and intestinal barrier function in mice induced by CYP by regulating intestinal flora imbalance^8^. Therefore, regulating intestinal flora, inflammation, and intestinal barrier may be a novel potential therapeutic strategy for the treatment of the intestinal injury.

Probiotics are considered a novel choice to reduce the side effects of chemotherapy on patients. Probiotics can maintain the balance of intestinal microorganisms and protect the intestinal integrity of epithelial cells by increasing the mucus layer and the expression of TJ^9^. *Bacillus coagulans*, as a probiotic, can stay in the gut for a longer time; it also has a strong adhesive ability and significant immunomodulatory effect^10^. It has been reported that some active components in the fermentation supernatant of *B. coagulans* can form a biological protective barrier in the human intestinal tract, promote the immune response of the digestive tract mucosa, and thus improve intestinal immunity^11^. In addition, *B. coagulans* 13002 can stimulate the growth of *Bifidobacterium* and *Lactobacillus* and reduce the intestinal side effects caused by cyclophosphamide^12^. Furthermore, *B. coagulans* TL3 can protect rats from inflammation caused by endotoxin and inhibit the reproduction of harmful bacteria by blocking the expression of the TLR4 pathway so as to enhance intestinal immunity^13^. Moreover, previous studies have shown that *B. coagulans* 13002 and fructooligosaccharides significantly reduce CYP-induced intestinal mucosal damage and improve immune function by regulating intestinal microflora^12^. In this study, we examined the effects of *B. coagulans* MZY531 (MZY531) on intestinal mucosal injury, inflammation, and intestinal microflora induced by CYP in mice and explored its protective mechanism. These data provide a theoretical basis for the development and utilization of *B. coagulans* and support its addition to functional foods to improve intestinal health.

## Materials and methods

### Preparation of bacterial strain

*B. coagulans* MZY531 is a probiotic strain isolated from naturally fermented kimchi and stored in China Center for Type Culture Collection (CCTCC, accession M2021622, Wuhan, China). *B. coagulans* MZY531 was inoculated in LB liquid medium and cultured in a constant temperature vibration incubator for 48 h (180 r/min, 50 °C). Then the culture medium was centrifuged (2000×*g*, 10 min) and washed three times with aseptic phosphate-buffered saline (PBS, pH 7.4) to remove the residual medium and collect bacteria. Next, the bacteria were resuspended in saline solution, and the concentration was adjusted to 1.0 × 10^9^ CFU/mL, which was stored at 4 °C for subsequent intragastric administration of mice.

### Animals and experimental design

Immunosuppressive model was induced by CYP according to previous study^14^. A total of 40 7-week-old female BALB/C mice were purchased from Changchun Yisi Experimental Animal Technology Co., Ltd. (Changchun, China). All mice were kept in a suitable environment with a temperature of 22 ± 1 °C, relative humidity of 50 ± 1%, and a light/dark cycle of 12 h, and had free access to water and food. All animal studies (including the mice euthanasia procedure) were done in compliance with the regulations and guidelines of the Jilin Academy of Agricultural Sciences institutional animal care and conducted according to the AAALAC and the IACUC guidelines.

**Figure 1:**
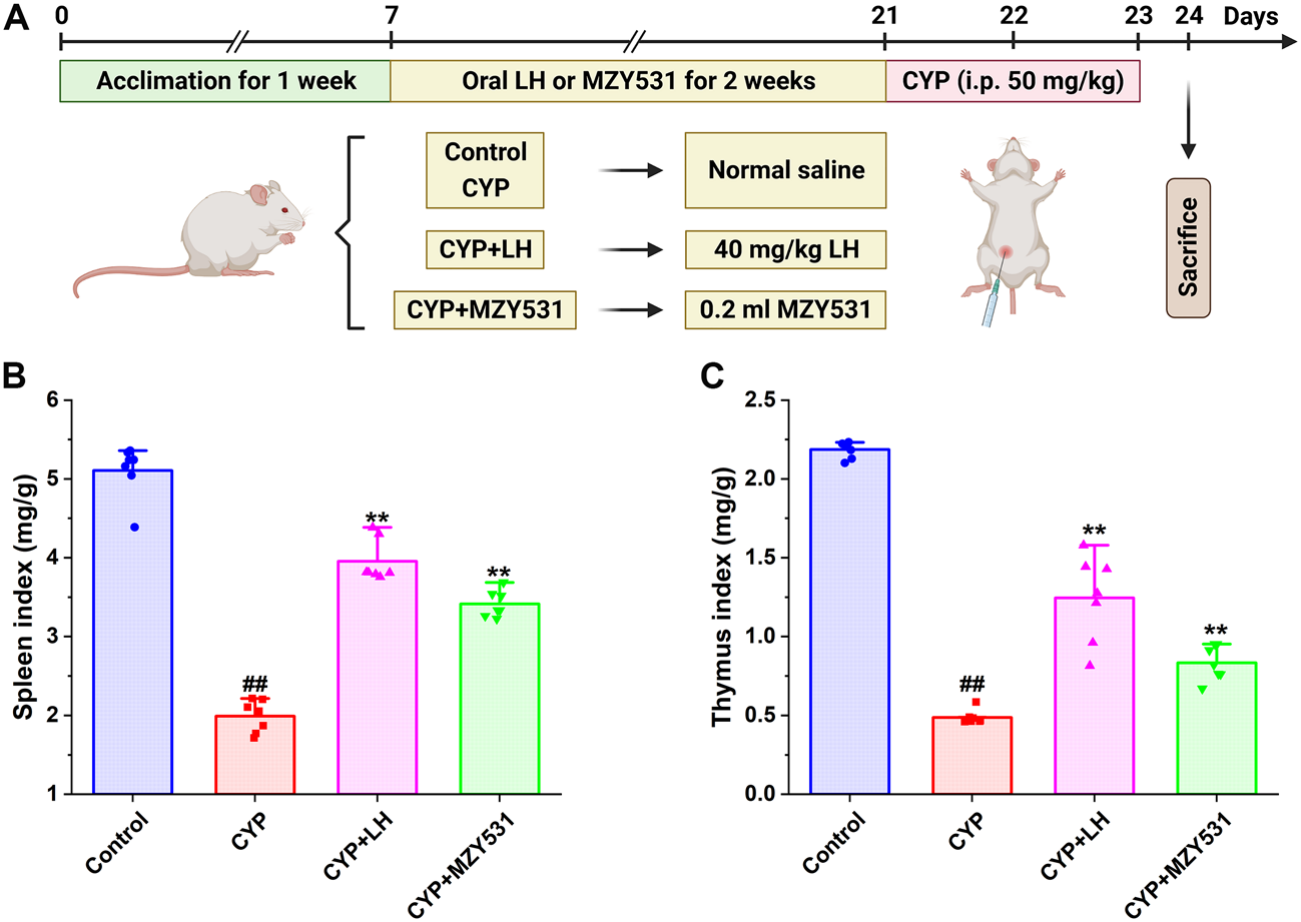
A schedule of experimental procedures (**A**) and effects of *B. coagulans* MZY531 on spleen (**B**) and thymus (**C**) index in immunosuppressed mice. All data were statistically analyzed using a one-way analysis of variance and Tukey multiple comparison. ^#^*P* < 0.05 and ^##^*P* < 0.01 *vs.* control group; *P < 0.05 and **P < 0.01 *vs.* CYP group.

The experimental animal protocol is shown in Fig. 1A. After 1 week of adaptation, the mice were randomly divided into four groups (n = 10 in each group): Control group, CYP group, CYP + LH group, and CYP + MZY531 group. The body weights of the mice were measured twice every week. The CYP + LH group was given 40 mg/kg levamisole hydrochloride (LH), the CYP + MZY531 group was given *B. coagulans* MZY531, and the Control and CYP groups were given the same dose of normal saline. All mice were given oral administration according to the volume of 0.1 mL/10 g for 14 days, once daily. The immunosuppression mouse model induced by CTX was established according to the previous method. CYP (50 mg/kg/days) was intraperitoneally injected into the mice in CYP, CYP + LH, and CYP + MZY531 groups on days 15 and 16. Control group was administered intraperitoneally with the same volume of physiological saline. After the last injection, the mice were starved for 24 h but given free access to water. The mice were sacrificed by cervical dislocation, and the jejunum, ileum, spleen, and feces were collected.

### Determination of immune organ index

Before the mice were killed, the final weight of the mice was recorded. Then, the thymus and spleen tissues were immediately dissected, washed in precooled normal saline at 4 °C, dried using filter paper, and weighed. The spleen and thymus index were calculated according to the following formula: spleen and thymus index = spleen and thymus weight (mg)/final weight (g)^15^.

### Determination of immune and inflammatory factors in the ileum

The ileum of mice was quickly collected and placed in an ice bath. An appropriate amount of ileum tissue was then selected and mixed with normal saline according to the proportion of 1:9 to prepare 10% tissue homogenate. Next, the homogenate was centrifuged (4000×*g*, 10 min) at 4 °C, and the supernatant was collected. The immunoglobulin (IgA, IgE, IgG, and IgM) and inflammatory factors (IL-2, IFN-γ, IL-4, and IL-10) concentrations were detected using ELISA kits according to the instructions of Jiangsu Enzymatic Biology Co., Ltd. (Jiangsu, China). The optical density (OD) value of the solution was measured at 450 nm using an automatic microplate reader.

### Pathological observation of jejunum

The fresh jejunum tissue was washed with normal saline, fixed in 4% paraformaldehyde for 48 h, embedded in paraffin, and prepared into 8-μm slices. Then, samples were stained with hematoxylin–eosin (H&E) for 5 min, dehydrated, and sealed with neutral glue. The pathological changes in the jejunum of each group were observed under light microscope (Nikon Corporation, Tokyo, Japan), and the villus length (V) and crypt depth (C) were recorded^16^. Moreover, the images were acquired at a magnification of × 25 and × 200 (Supplementary Information).

### Western blotting

The ileal tissue was lysed by RIPA kit, and the supernatant was collected. The BCA kit determined the total protein concentration in the supernatant. Samples were then mixed with the protein sample buffer at 1:1 and boiled in a water bath for 8 min to collect the proteins, which were then separated by sodium dodecyl sulfate–polyacrylamide gel electrophoresis (SDS-PAGE) and transferred to polyvinylidene fluoride (PVDF) membrane. The membrane was blocked for 60 min in TBST solution containing 3% bovine serum albumin (BSA) and then incubated with rabbit anti-ZO-1, Occludin, Claudin-1, TLR4, MyD88, NF-κB, IKBα, and β-actin overnight at 4 °C. Samples were then incubated with horseradish peroxidase (HRP) labeled secondary antibody for 60 min at room temperature. Samples were then washed with TBST three times, and the protein expression was detected by an enhanced chemiluminescence reagent. The gray values of the bands were detected by Image Quant LAS 4000 (Shanghai, China) and standardized by β-actin.

### Gut microbial analysis

Fresh fecal samples of the cecum were immediately frozen in liquid nitrogen and stored at − 80 °C. QIAamp Fast DNA kit was used to extract total DNA from feces. According to the previous study^17^, the V3 and V4 regions of 16S rDNA were amplified by universal primers using polymerase chain reaction (PCR). Then the amplified products were sequenced by Illumina MiSeq, and the sequences of high quality with 97% similarity were incorporated into a taxon on QIIME software, and the diversity of gut microbiota was analyzed. The Chao1, Shannon, Simpson, and Pielou-e indices were used to investigate α diversity. The principal coordinate method of weighted UniFrac phylogenetic distance matrix was used to analyze β diversity; the relative abundance at the gate level was used to indicate the difference in bacterial colony structure among groups, and the heat map analysis showed the difference in different microorganisms at the genus level. In addition, Spearman’s analysis revealed the correlation between gut microbiota and immune and inflammatory levels in mice. The original data and sequencing sample data obtained in this study can be obtained from the National Center for Biotechnology Information (NCBI) database with the registration number: PRJNA884309.

### Statistical analysis

All the experimental data were expressed as mean ± standard deviation (SD). SPSS20.0 and Origin8.0 were used for data processing and analysis. The overall significant difference was evaluated by single factor analysis of variance (ANOVA) and Tukey multiple comparisons. A *P* value < 0.05 was considered to be statistically significant.

### Institutional review board statement

The animal experiment procedures were approved by the Committee of Animal Experimental Ethical Inspection of Laboratory Animal Centre, Yanbian University (approved number: SCXK-2020-0001).

### ARRIVE guidelines

All the research methods contained in the manuscript are carried out in accordance with the requirements of ARRIVE.

## Results

### *B. coagulans* MZY531 increases the immune organ index of intestinal injury mice

As shown in Fig. 1, the intervention of CYP significantly decreased the spleen (Fig. 1B) and thymus (Fig. 1C) index of mice compared with the control group, while the treatment of LH and *B. coagulans* MZY531 significantly increased the spleen and thymus index (all *P* < 0.01). After *B. coagulans* MZY531 treatment, the spleen and thymus index increased by 71.36% and 69.39%, respectively, compared with the CYP group (all *P* < 0.01). These results indicate that *B. coagulans* MZY531 could effectively alleviate immune organ atrophy induced by CYP. Additionally, the spleen and thymus indexes of the *B. coagulans* MZY531 group were higher than in the CYP group, with the indexes being close to those of the LH group. These findings suggest that *B. coagulans* MZY531 plays a crucial role in preventing the atrophy of immune organs.

### *B. coagulans* MZY531 increases the level of immune protein in the ileum of intestinal injury mice

**Figure 2:**
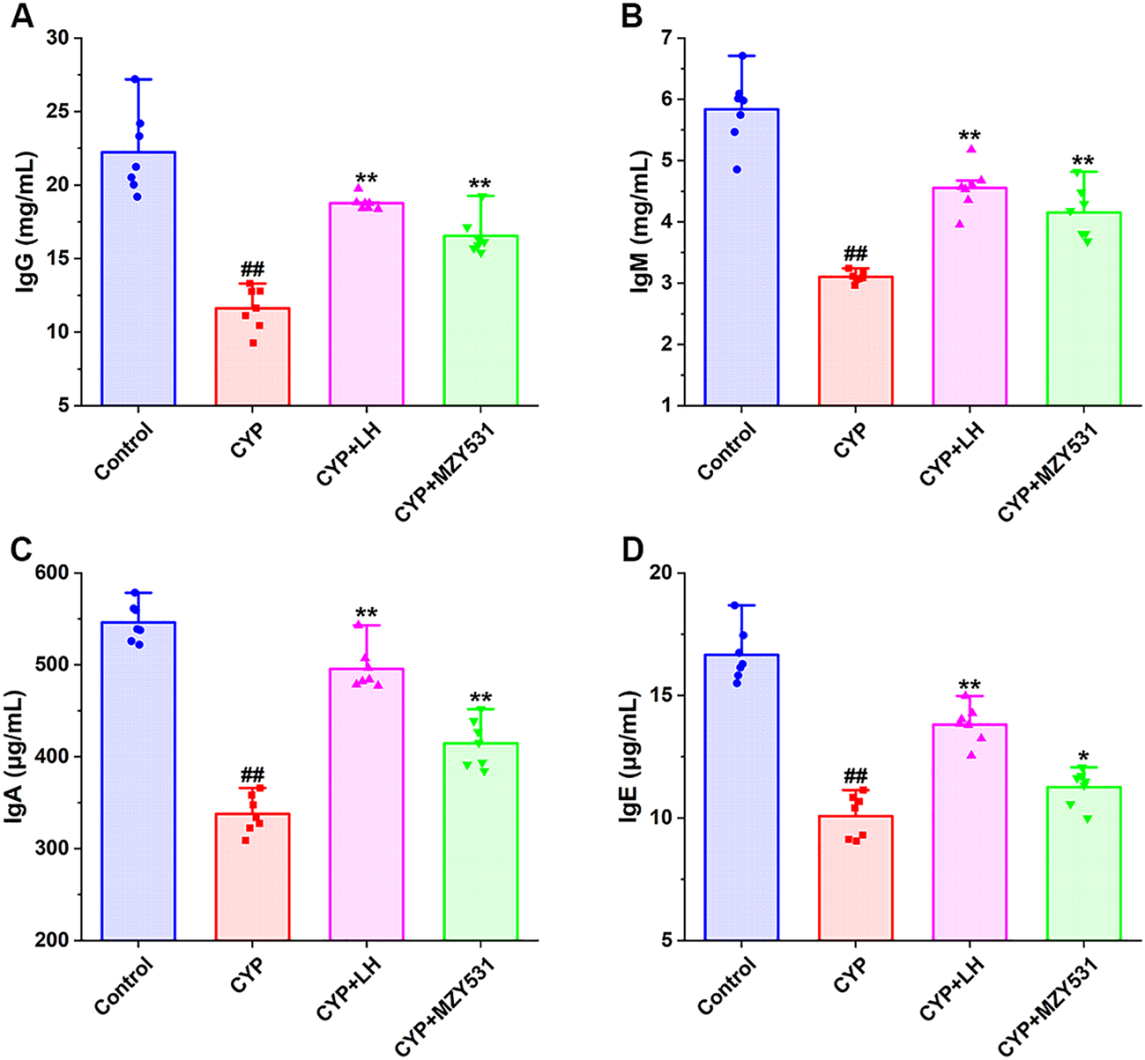
Effects of *B. coagulans* MZY531 on the levels of immune proteins level in ileum of mice with intestinal injury induced by CYP. IgG (**A**); IgM (**B**); IgA (**C**) and IgE (**D**). All data were statistically analyzed using a one-way analysis of variance and Tukey multiple comparison. ^#^*P* < 0.05 and ^##^*P* < 0.01 *vs.* control group; *P < 0.05 and **P < 0.01 *vs.* CYP group.

The results showed that (Fig. 2A–D), the induction of CYP significantly decreased the levels of IgG, IgM, IgA, and IgE by 47.75%, 46.92%, 38.15%, and 39.50%, respectively, compared with the control group (all *P* < 0.01). The levels of IgG, IgM, IgA, and IgE in the *B. coagulans* MZY531 treatment were significantly higher than those in the CYP group (*P* < 0.05), approaching the values of the control group. After the *B. coagulans* MZY531 treatment, the levels of IgG and IgM were similar to those of the positive control LH group (*P* > 0.05). These results showed that the treatment of *B. coagulans* MZY531 could reverse the decrease of immune protein level induced by CYP and improve the immunity of mice.

### *B. coagulans* MZY531 increases the level of anti-inflammatory cytokines in the ileum of intestinal injury mice

**Figure 3:**
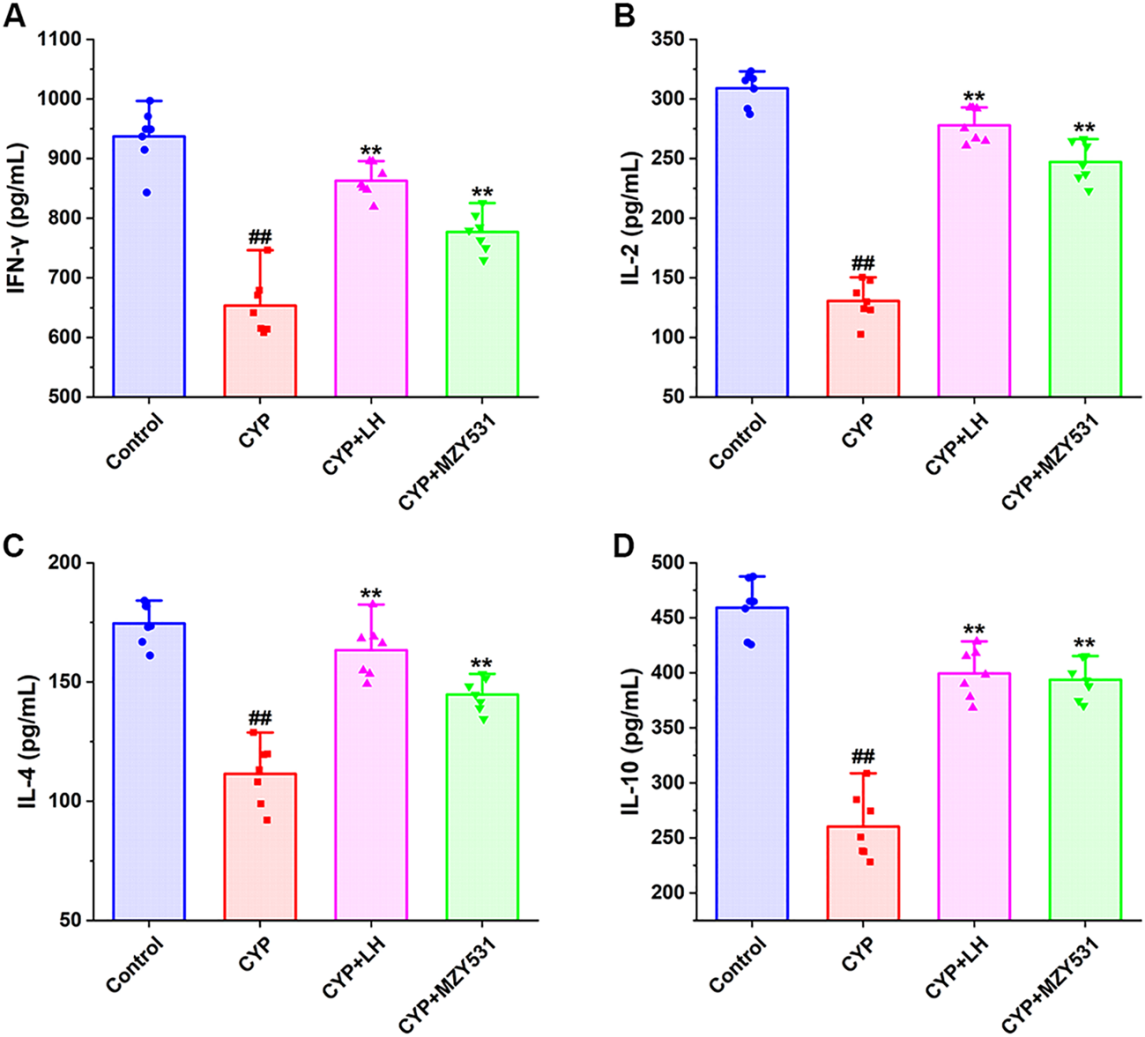
Effects of *B. coagulans* MZY531 on the levels of anti-inflammatory factors in ileum of immunosuppressed mice. IFN-γ (**A**); IL-2 (**B**); IL-4 (**C**) and IL-10 (**D**). All data were statistically analyzed using a one-way analysis of variance and Tukey multiple comparison. ^#^*P* < 0.05 and ^##^*P* < 0.01 *vs.* control group; *P < 0.05 and **P < 0.01 *vs.* CYP group.

As shown in Fig. 3A–D, the levels of IFN-γ, IL-2, IL-4, and IL-10 in the CYP group were significantly lower than those in the control group (*P* < 0.05), indicating that CYP could significantly inhibit the production of anti-inflammatory cytokines. Compared with the CYP group, the treatment of *B. coagulans* MZY531 significantly increased the levels of IFN-γ, IL-2, IL-4, and IL-10 by 18.86%, 89.03%, 29.81%, and 51.14%, respectively (*P* < 0.05). The above results also showed that *B. coagulans* MZY531 could improve the anti-inflammatory ability of CYP-induced intestinal injury model mice. Furthermore, *B. coagulans* MZY531, in particular, significantly improved the secretion of IL-10. These results indicated that *B. coagulans* MZY531 could improve inflammatory responses by increasing the secretion of anti-inflammatory cytokines in ileum of CYP-induced immunosuppressed mice.

### *B. coagulans* MZY531 improves the histomorphological changes of jejunum in intestinal injury mice

**Figure 4:**
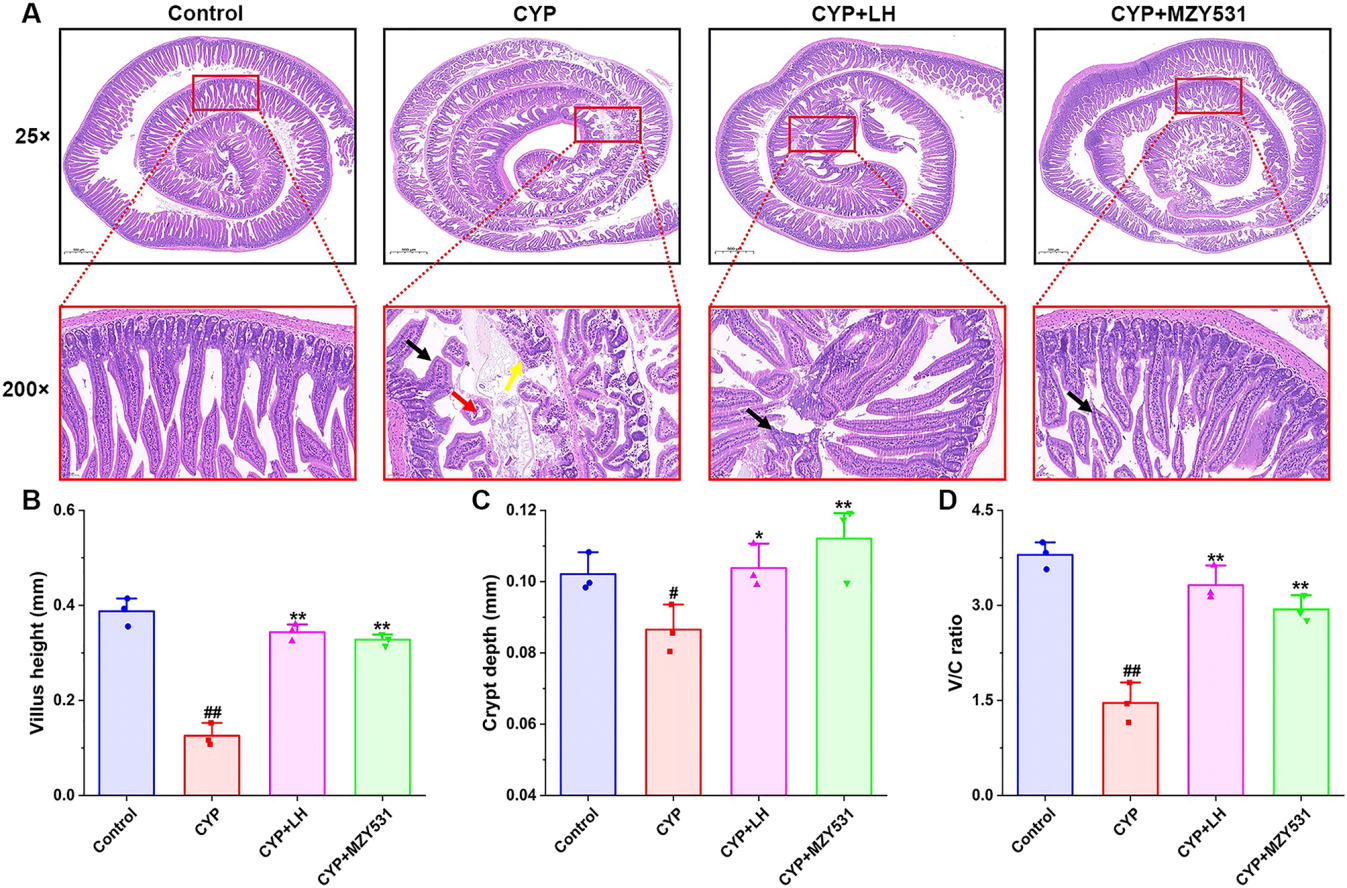
Effects of *B. coagulans* MZY531 on the histomorphological changes of jejunum in immunosuppressed mice. The pathological changes of jejunum (magnification × 25 and × 200) (**A**). Black arrows indicated the shorterning of intestinal villi, yellow arrows indicated exposed lamina propria, and black arrows indicated mild edema and enlarged spaces. Villus height (**B**), Crypt depth (**C**). The ratio of villus height to crypt depth (**D**). All data were statistically analyzed using a one-way analysis of variance and Tukey multiple comparison. ^#^*P* < 0.05 and ^##^*P* < 0.01 *vs.* control group; *P < 0.05 and **P < 0.01 *vs.* CYP group.

The results of HE staining of the jejunum of mice are shown in Fig. 4A, while no obvious pathological changes in jejunum were found in the blank group. In the CYP group, the jejunal villi were shortened and exfoliated (black arrow), the intestinal epithelium of the local mucous layer was missing, the lamina propria was exposed (yellow arrow), slight edema could be seen locally, and the gap between the intestinal epithelium and lamina propria was seen (red arrow). However, the villi length increased, and the intestinal epithelial structure was significantly recovered in LH and *B. coagulans* MZY531 groups. In addition, the treatment of MZY531 significantly increased villus length (Fig. 4B), crypt depth (Fig. 4C), and the V/C ratio (Fig. 4D), which were 153.85%, 26.44%, and 101.37%, respectively, higher than those of the CYP group (all *P* < 0.01). These results also suggested that *B. coagulans* MZY531 could improve the pathological intestinal damage in CYP-induced intestinal injury model mice.

### *B. coagulans* MZY531 improves intestinal barrier function in intestinal injury mice

**Figure 5:**
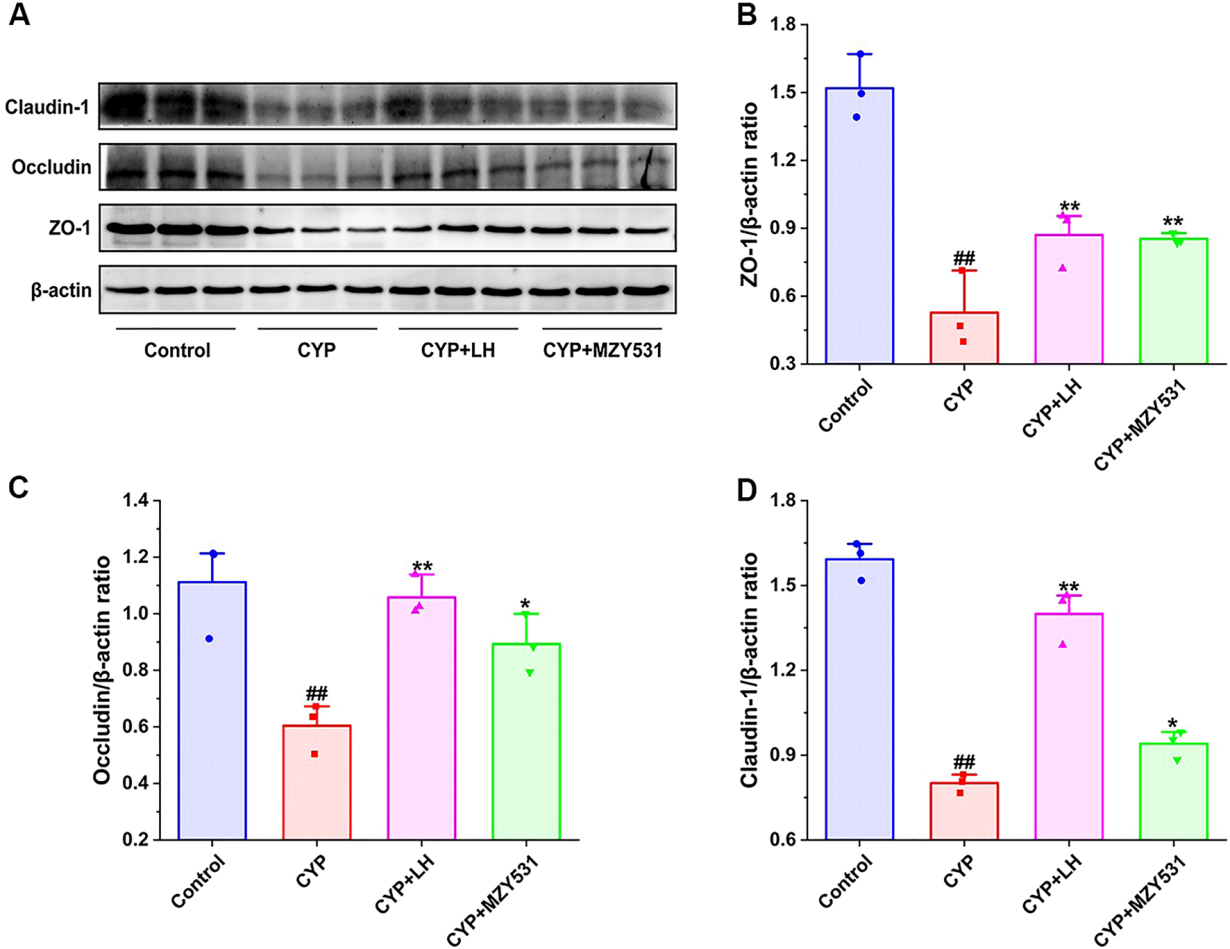
Effects of *B. coagulans* MZY531 on ZO-1 intestinal barrier pathway in ileum of immunosuppressed mice. The protein expression of Claudin-1, Occludin and ZO-1 in the ileal were detected by western blot (**A**). The ratios of ZO-1/β-actin (**B**), Occludin/β-actin (**C**) and Claudin-1/β-actin (**D**) protein bands for each region were quantified using densitometry and presented in the graph. All data were statistically analyzed using a one-way analysis of variance and Tukey multiple comparison. ^#^*P* < 0.05 and ^##^*P* < 0.01 *vs.* control group; *P < 0.05 and **P < 0.01 *vs.* CYP group.

The effect of *B. coagulans* MZY531 on proteins (ZO-1, occludin, and claudin-1) of the intestinal barrier pathway in the ileum of mice was evaluated by Western blotting. As shown in Fig. 5, compared with the blank group, the induction of CYP significantly decreased the protein levels of ZO-1, occludin, and claudin-1 (all *P* < 0.05). Compared with CYP group, Intervention with *B. coagulans* MZY531 significantly increased the protein expression of ZO-1, occluding, and claudin-1 by 61.87% (*P* < 0.01), 47.80% (*P* < 0.05), and 17.32% (*P* < 0.05), respectively. Based on this result, *B. coagulans* MZY531 could repair the intestinal barrier damage induced by CYP in mice.

### *B. coagulans* MZY531 inhibits the level of inflammation in intestinal injury mice

**Figure 6:**
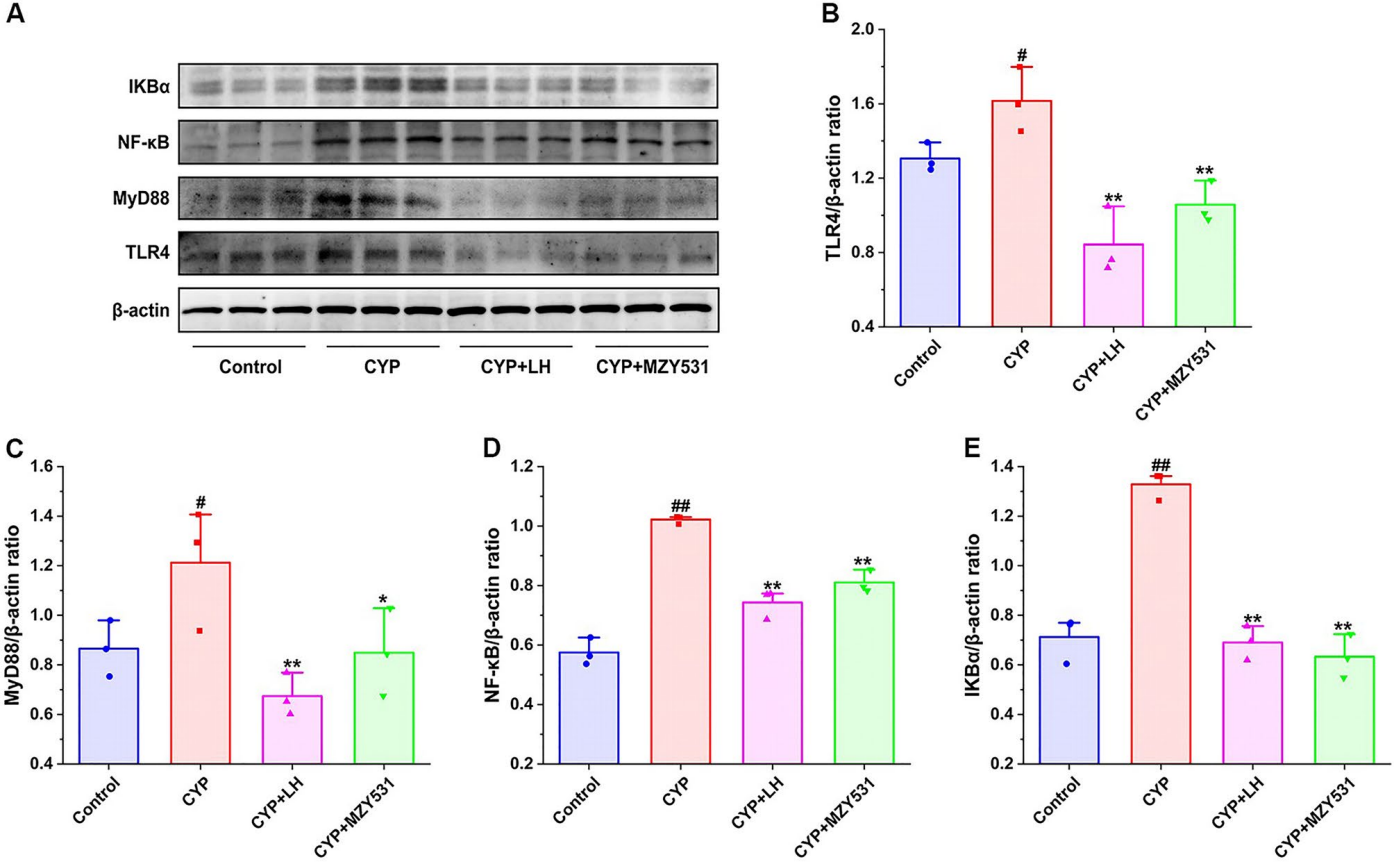
Effects of *B. coagulans* MZY531 on TLR4/MyD88 inflammatory pathway in ileum of immunosuppressed mice. The protein expression of IKBα, NF-κB, MyD88 and TLR4 in the ileal were detected by western blot (**A**). The ratios of TLR4/β-actin (**B**), MyD88/β-actin (**C**), NF-κB/β-actin (**D**) and IKBα/β-actin (**E**) protein bands for each region were quantified using densitometry and presented in the graph. All data were statistically analyzed using a one-way analysis of variance and Tukey multiple comparison. ^#^*P* < 0.05 and ^##^*P* < 0.01 *vs.* control group; *P < 0.05 and **P < 0.01 *vs.* CYP group.

The results presented in Fig. 6 shows that the expression of TLR4 inflammatory pathway protein in mouse jejunum. The induction of CYP significantly upregulated the levels of TLR4, MyD88, NF-κB, and IKBα. On the contrary, the treatment of LH and *B. coagulans* MZ531 significantly inhibited the expression of TLR4, MyD88, NF-κB and IKBα, and the level of MZY531 group was down-regulated by 34.55% (*P* < 0.01), 29.95% (*P* < 0.05), 20.71% (*P* < 0.01), and 52.36% (*P* < 0.01) compared with CYP group, respectively. Thus, these results revealed that the treatment of *B. coagulans* MZY531 markedly resisted the expression of intestinal inflammation induced by CYP through downregulation of TLR4/MyD88/NF-κB inflammatory signaling pathways.

### *B. coagulans* MZY531 remodels the intestinal microflora of intestinal injury mice

**Figure 7:**
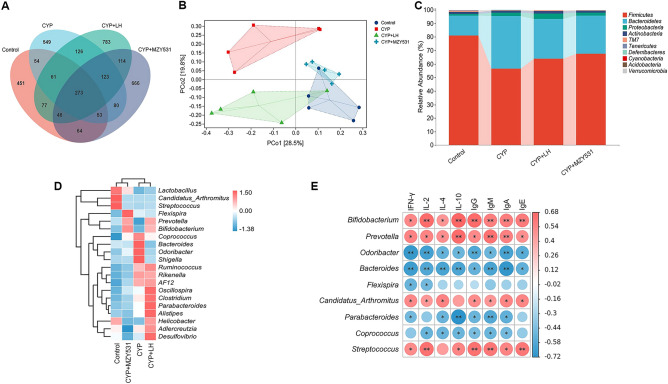
Effects of *B. coagulans* MZY531 on the changes of gut microbiota in immunosuppressed mice. Venn diagram (**A**); PCoA analysis (**B**). The species compositions analysis at phylum level (**C**). The heat map at genus level (**D**). The correlation analysis between gut microbiota and intestinal immune proteins and anti-inflammatory factors at genus level by Spearman analysis (**E**). ^#^*P* < 0.05 and ^##^*P* < 0.01 *vs.* control group; *P < 0.05 and **P < 0.01 *vs.* CYP group.

We evaluated the effect of *B. coagulans* MZY531 on gut microbiota by 16S rRNA high-throughput sequencing. The results of α diversity showed that (Table 1), the indexes of Chao1, Shannon, Simpson, and Pielou-e in the *B. coagulans* MZY531 group were significantly higher than those in the CYP group (all *P* < 0.01), which suggested that the intervention of *B. coagulans* MZY531 increased the richness and diversity of gut microbiota. Venn diagram (Fig. 7A) further showed that the four groups shared 273 OTU, while the number of unique OTU in the blank group, CYP group, LH group, and MZY531 group was 451, 549, 783, and 666, respectively, indicating that the treatment of *B. coagulans* MZY531 increases the number of OTU induced by CYP. In addition, in PCoA analysis (Fig. 7B), the CYP group was far away from the blank group, LH group, and MZY531 group, while the treatment of LH and *B. coagulans* MZY531 made the diversity of gut microbiota of mice more inclined to the blank group. In order to further evaluate the specific changes in gut microbiota, we investigated the relative abundance of gut microbiota (Fig. 7C) at the gate level. We found that *B. coagulans* MZY531 treatment increased *Firmicutes* but decreased *Bacteroidetes* abundance. In genus-level thermographic analysis (Fig. 7D), the intervention of *B. coagulans* MZY531 increased the abundance of probiotic, including *Lactobacillus*, *Prevotella* and *Bifidobacterium*, and decreased the level of harmful bacteria *Odoribacter* a*nd Shigella.* These results showed that the intervention of *B. coagulans* MZY531 could reshape the structure of gut microbiota, increase the abundance of probiotics, and reduce the level of pathogenic bacteria.

**Table 1:** Effects of MZY531 on α-diversity of gut microbiota in mice with intestinal injury induced by CYP.

Groups	Chao1	Shannon	Simpson	Pielou_e
Control	516.75 ± 112.82**	5.57 ± 0.66**	0.9326 ± 0.027**	0.6286 ± 0.05*
CYP	341.79 ± 82.56^##^	4.33 ± 0.52^##^	0.8397 ± 0.04^##^	0.5254 ± 0.05^#^
CYP + LH	548.66 ± 39.71**	5.83 ± 0.49**	0.9488 ± 0.024**	0.651 ± 0.05**
CYP + MZY531	615.62 ± 72.18**	6.00 ± 0.41**	0.9516 ± 0.02**	0.658 ± 0.04**

All data were statistically analyzed using a one-way analysis of variance and Tukey multiple comparison.

^#^*P* < 0.05 and ^##^*P* < 0.01 *vs.* control group; *P < 0.05 and **P < 0.01 *vs.* CYP group.

Next, Spearman analysis was used to analyze the correlation between single strains of gut microbiota and immune and anti-inflammatory proteins in mice (Fig. 7E), revealing that *Prevotella* and *Bifidobacterium* were positively correlated with immune proteins, including IgG, IgM, IgA, and IgE, and anti-inflammatory factors, including IFN-γ, IL-2, IL-4, and IL-10, while *Bacteroidetes* and *Odoribacter* was negatively correlated with immune proteins and anti-inflammatory factors. These results further reveal that the intervention of *B. coagulans* MZY531 could increase the immunity and anti-inflammatory ability of mice by increasing the abundance of probiotics.

## Discussion

CYP is a widely used chemotherapeutic drug for cancer treatment. Yet, CYP can seriously damage the body’s immunity and induce the disorder of intestinal microflora, thus increasing the risk of immune deficiency and intestinal injury diseases^18^. It has been found that probiotics can improve the function of intestinal microflora by regulating the value of specific microflora in the intestinal tract, thus having a beneficial effect on the body^19^. Therefore, we speculate that probiotics may be a new therapeutic way to alleviate CYP-induced intestinal injury. This study focused on the protective effect of *B. coagulans* MZY531 on intestinal injury induced by CYP.

As important immune organs, spleen and thymus have an important role in regulating systemic immune function^20^. Our study showed that the intervention of *B. coagulans* MZY531 significantly increases the spleen and thymus index of mice induced by CYP. Coincidentally, Awad et al*.* reported that the use of probiotics increases the spleen and thymus index of broilers^21^; similar results were obtained by Kabir et al.^22^. In addition, the occurrence of immune dysfunction seems to be regulated by IFN-γ and IL-4 levels, and the decrease of their levels may lead to impaired immune function^23^. Studies have shown that CYP, as an effective immunosuppressant, can induce the decrease of IFN-γ and IL-4 levels, thus destroying immune homeostasis and leading to immunosuppression^24^. It is worth noting that *B. coagulans* MZY531 treatment significantly increases the levels of IFN-γ and IL-4 and increases the expression of immune-related cytokines, including IgG, IgM, IgA, and IgE. Furthermore, Bomko et al*.* found that *B. coagulans* had normalised both the quantitative parameters of the immune system and the cells’ functional activity by decreasing the level of immune-related cytokines^25^. This study further suggests that treating *B. coagulans* MZY531 can resist the immune function damage induced by CYP by improving the function of immune organs and the level of immune protein.

As the main food digestive organ most easily affected by foreign antigens or microorganisms, the intestinal tract is the first line of defense against pathogenic microorganisms^26^. A harmful environment may induce oxidation and inflammation in the intestinal tract, damaging intestinal mucosal. Studies have shown that CYP can induce the shortening and shedding of intestinal villi and, in turn, lead to intestinal mucosal damage^27^. In this study, the pathological results showed that the treatment of *B. coagulans* MZY531 significantly reduces the shedding of intestinal villi, increases the crypt depth, and reduces the edema of intestinal endothelial cells, which indicates that the intervention of *B. coagulans* MZY531 could alleviate the intestinal mucosal injury induced by CYP. It is worth noting that the impairment of intestinal mucosal barrier function seems to be the main inducing factor of intestinal mucosal injury^28^. Therefore, increasing intestinal barrier function seems to be a good means to prevent or treat intestinal injury. As intestinal tight junction proteins, ZO-1, occludin, and claudin-1 have an important role in maintaining intestinal barrier permeability and constitute intestinal mucosal barrier with intestinal epithelial cells^29,30^. According to previous studies, *B. coagulans* SCC-19 improves the intestinal barrier of carp induced by heavy metal cadmium (Cd) by up-regulating the mRNA expression of ZO-1, occluding, and claudin-1^31^. In addition, Zhou et al*.* confirmed that NCU116 extracellular polysaccharides of *Lactobacillus plantarum* could increase the expression of the ZO-1 tight junction protein pathway by promoting the binding of STAT3 to occludin and ZO-1 promoters, thus repairing the intestinal mechanical barrier function induced by dextransodiumsulfate (DSS)^32^. Importantly, our study also found that MZY531 activates the expression of ZO-1, occluding, and claudin-1 proteins, thus restoring intestinal mucosal barrier function. Therefore, we speculate that MZY531 may improve intestinal permeability by activating the expression of intestinal tight junction protein, thus resisting intestinal mucosal injury induced by CYP.

In recent years, increasing evidence has shown that intestinal injury can also induce intestinal leakage (leaky gut), causing bacteria and their metabolites to translocate to the blood, releasing inflammatory factors, including LPS and TNF-α^33^. In addition, the accumulation of pro-inflammatory mediators can break the balance of anti-inflammatory and pro-inflammatory factors and further aggravate the inflammatory cascade and intestinal injury^34^. As a specific anti-inflammatory factor, IL-10 has an important role in enhancing the anti-inflammatory ability of the body^35^. It has been reported that probiotics can reduce advocated inflammatory expression by activating IL-10-mediated immune pathways^36^. Interestingly, similar results were obtained in this study. Therefore, enhancing the expression of the anti-inflammatory factor IL-10 may be one of the keys to controlling intestinal inflammation. Nevertheless, some studies have reported that the expression of IL-10 is affected by the TLR4 pathway. As a type I transmembrane protein expressed on the cell membrane, TLR4 has an important role in regulating the balance of inflammation^37^. The NF-κB pathway is a downstream signal transduction pathway dependent on TLR4/MyD88 pathway. When the body is in normal homeostasis, NF-κB will bind to I-κB and remain static^38^. However, external stimulation can activate the expression of the TLR4/MyD88 pathway and further induce the activation of the I-κB complex, thus regulating the expression of target genes, including TNF-α, IL-1β, IL-6, and IL-10^39^. It is reported that *B. coagulans* TL3 inhibits intestinal inflammation induced by LPS through TLR4/MyD88, which suggests that the TLR4/MyD88 signal pathway may be the signal transduction mechanism of *B. coagulans* inhibiting intestinal inflammation expression^13^. In addition, some scholars have reported that the combined treatment of several probiotics, including *B. coagulans*, suppresses DSS-induced colitis by up-regulating the level of IL-10^40^. Interestingly, it has been reported that E5564, an antagonist of TLR4, can competitively bind to TLR4-MD2, further inhibit the activation of downstream NF-κB and promote the release of anti-inflammatory factor IL-10^41^. In this study, we found that MZY531 down-regulates the expression of the TLR4/MyD88 pathway and increases the level of IL-10. This further suggests that *B. coagulans* MZY531 may be an inhibitor of TLR4, i.e., it increases the level of IL-10 in the intestine of mice by inhibiting the TLR4/MyD88 pathway, thus improving the anti-inflammatory ability of mice and finally improving the intestinal inflammatory injury induced by CYP.

**Figure 8:**
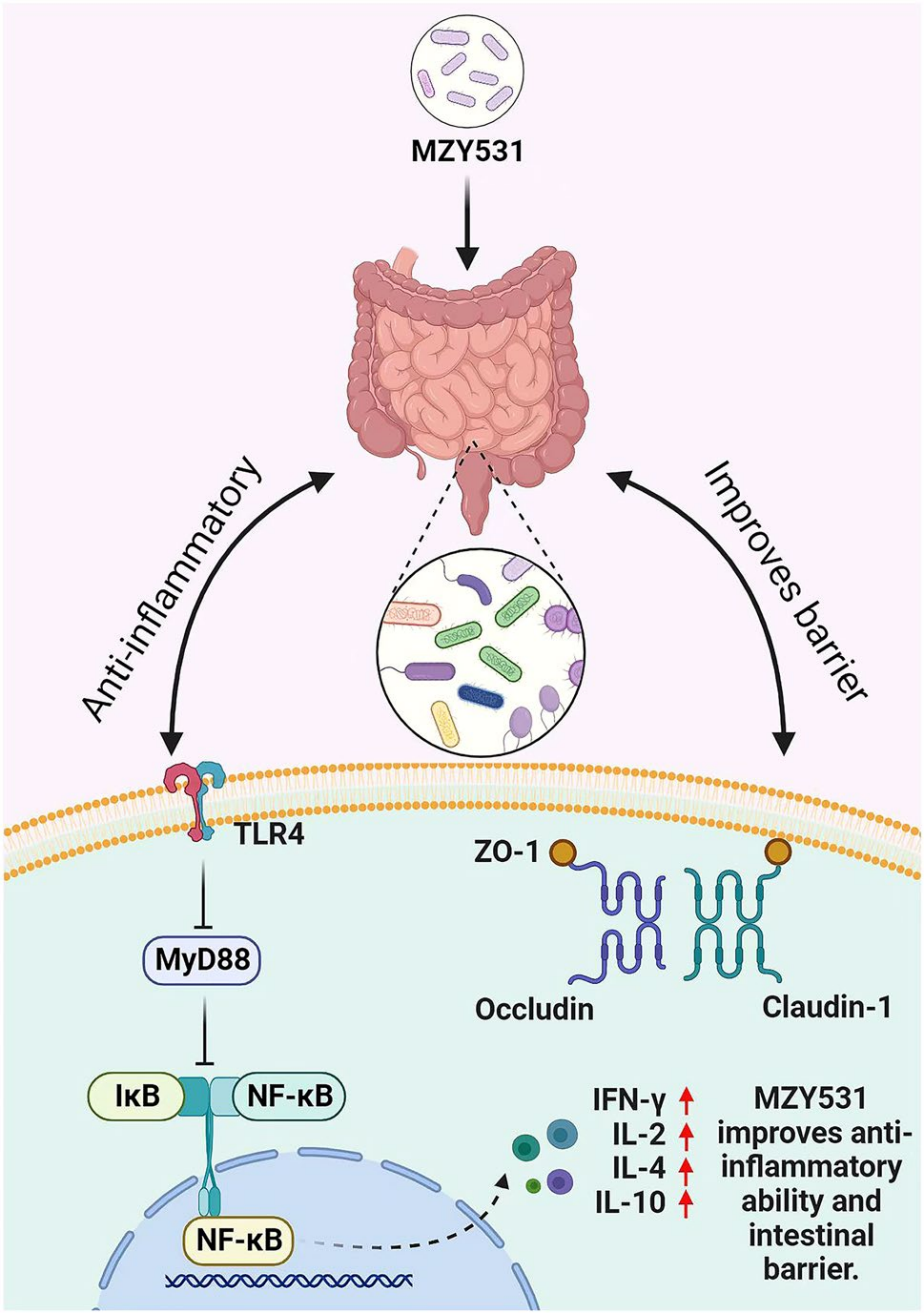
Schematic illustration showing the mechanisms of *B. coagulans* MZY531 alleviates intestinal mucosal injury in immunosuppressive mice.

*B. coagulans* can regulate the disorder of intestinal microflora, which has a beneficial effect on the host^42^. Studies have shown increased abundance and diversity of probiotics in feces collected from elderly taking *B. coagulans* GBI-30 and 6086 for 28 days^43^. Our study also obtained consistent results; we found that the intervention of *B. coagulans* MZY531 increases the total number of bacteria in the feces of mice with intestinal injury induced by CYP, which also indicates that *B. coagulans* MZY531 restores the abundance of intestinal flora. In addition, we also found that *B. coagulans* MZY531 can increase the abundance of probiotics (*Bifidobacterium, Prevotella* and *Firmicutes*) and reduce the number of bacteria causing inflammation (*Bacteroides* and *Shigella*). Of note, it has also been reported that taking *B. coagulans* 13002 increases intestinal damage induced by cyclophosphamide by increasing the abundance of probiotics^12^. In addition, Xie et al*.* reported that taking *L. plantarum* NCU116 may increase the number of *Bifidobacteria* in the feces of mice, and further reduce the disorder of gut microbiota, thus improving the intestinal mucosal damage induced by CYP^8^. This indirectly confirms that *B. coagulans* MZY531 has an important role in improving the intestinal injury induced by CYP. Interestingly, we examined the correlation between intestinal flora and intestinal immune function and anti-inflammatory ability by Spearman analysis, finding that *Bifidobacterium* and *Prevotella* were positively correlated with immune proteins and anti-inflammatory factors in the intestinal tract; similar conclusions were reached by Li et al.^44^. This further suggests that the intervention of *B. coagulans* MZY531 can improve the immune and anti-inflammatory ability of the intestine by increasing the abundance of probiotics in the intestine (Fig. 8), thus reducing the intestinal injury caused by intestinal inflammation and immunosuppression caused by CYP.

## Conclusion

To sum up, *B. coagulans* MZY531 treatment improves intestinal barrier function and inflammatory expression in CYP-induced immunosuppressive mice, and its possible mechanism is related to the ZO-1 intestinal barrier pathway and TLR4/MyD88 inflammatory pathway. In addition, *B. coagulans* MZY531 also improves the disorder of intestinal microflora by increasing the abundance of probiotics in the intestine and further improving the immune function and anti-inflammatory ability of mice. Therefore, this study provides a new research idea for treating intestinal injury in CYP-induced immunosuppressive mice and a solid theoretical basis for the development and utilization of *B. coagulans*.

## Supplementary Information

Supplementary Information.

## Data Availability

The data presented in this study are available on request from the corresponding author.
