# Supplementary material for: Bacillus coagulans MZY531 alleviates intestinal mucosal injury in immunosuppressive mice via modulating intestinal barrier, inflammatory response, and gut microbiota
Source: Sci Rep. 2023 Jul 10;13:11181. doi: 10.1038/s41598-023-38379-0 (PMC10333327; doi:10.1038/s41598-023-38379-0)
Supplement: Supplementary file 1 — Supplementary Information. [file 41598_2023_38379_MOESM1_ESM.doc]

Western blotting

Claudin-1

Occludin

ZO-1

β-actin

IKBα

NF-κB

MyD88

TLR4

β-actin

hematoxylin-eosin (H＆E) staining

Control

CYP

CYP+LH

CYP+MZY531
